# How Far Do Stomatologic Indications Warrant Constitutional Treatment?

**Published:** 1903-05

**Authors:** Eugene S. Talbot


### HOW FAR DO STOMATOLOGIC INDICATIONS WARRANT CONSTITUTIONAL TREATMENT?

BY EUGENE S. TALBOT, M.D., D.D.S.

THE alveolar process as a transitory structure through its relations to the trophic and vasomotor nerves is very susceptible to systemic impressions. The alveolar processes are terminal structures. The nerves and arteries passing through the alveolar process stop when in contact with the roots of the teeth, which fact, together with the transitory nature of the process, makes it doubly susceptible to the influence of interstitial gingivitis.

The alveolar processes and gums are frequently the structures first involved in disease. The most decided instances where deep-seated morbid states find expression in changes of the gums and alveolar processes are those due to lead, mercurial, and brass poisoning, anæmia, syphilis, diabetes, tuberculosis, renal disorders, scorbutus, etc.; in these the local stomatologic state is necessary for a complete diagnosis. In such cases local irritation or tissue nutritional disturbance from constitutional causes produces local tissue change by mechanic and chemic action (lactic and uric acid irritation from auto-intoxication and drug poisoning). Through such action blood strain increases and capillary dilatation results. The capillaries become crowded with blood-corpuscles which accumulate along the walls of the blood-vessels, to which they seemingly adhere. The accumulations of small round cells occur in the submucous tissue, the spaces of which are filled with inflammatory exudate. The papillæ become enlarged, the epithelial layer becomes hyperplastic, the gum tissues swell, become intensely crimson, and bleed upon the slightest touch. If the irritant factor be temporary in character and local in action, slight gingivitis results. If the irritation be permanent, the gingivitis becomes chronic, and, extending into the fibrous tissue below, becomes interstitial in type. The extent of this latter inflammation depends upon the nature of the irritation. If the irritation be located upon the side of one tooth, the inflammation extends to the fibrous tissue along the course of the blood-vessels. It may be in line with the peridental membrane, the periosteum, or in direct line with the alveolar process. Interstitial inflammation (should one or more teeth be involved) extends not only through the peridental membrane, but likewise to the periosteum and alveolar process, since the capillaries in surrounding structures are involved. The inflammation extends into the alveolar process through the Haversian canals and the blood-vessels of Von Ebner by way of the periosteum and peridental membrane. Irritation thereon resultant causes absorption of the alveolar process by (*a*) halisteresis, (*b*) Volkmann's perforating canal absorption, (*c*) lacunar absorption (osteoclasis). Interstitial inflammation continues as long as the irritation remains or until the tooth or teeth exfoliate. Occasionally bone necrosis results. Previous irritations often produce osteomalacia and trophic changes, thus assisting pathologic progress. Endarteritis obliterans, alveolar process absorption, pyorrhœa alveolaris, and, finally, calcic deposit may complicate these conditions. Endarteritis obliterans may result through thickening of one or all coats, thus interfering with the blood circulation. This results from the toxic element in the blood and degeneration of the peripheral nerve. Pus infection then readily occurs. Circulation being impaired, the calcic deposits from the blood do not occur directly, but around the Haversian canals (halisteresis) and through the vessels of Von Ebner; bone absorption is rapidly occurring; calcic material from the alveolar process is thereby deposited directly upon the roots of the teeth. Such are the stomatologic indications which warrant constitutional treatment.

The effect of drugs producing inflammation and destruction of the gums and alveolar processes depends upon the etiologic moment, which is made up of the general constitutional character of the patient as well as his condition at the time of drug administration. As the alveolar process in certain toxæmias (due either to drug action, to auto-intoxication, or to nutritional disturbance) serve for elimination purposes, of necessity products eliminated tend to pass through these processes when elimination elsewhere is insufficient. When elimination has once taken this direction a predisposition to such elimination thereby occurs. If in early life scorbutus, peliosis rheumatica, phosphorus poisoning, or any condition producing sialorrhœa have occurred, in later life drug administration is peculiarly apt to set up inflammatory irritation. Drug administration is hence apt to have untoward irritant effects in such subjects. These, as Rabelais long ago pointed out, may result as notoriously in the case of mercury, in interstitial gingivitis with tooth exfoliation. The same conditions appear as the untoward effects of other drugs.

